# Editorial for the Special Issue “Glioblastoma: What Do We Know?”

**DOI:** 10.3390/cells15020200

**Published:** 2026-01-21

**Authors:** Shan Ping Yu

**Affiliations:** School of Medicine, Emory University, Atlanta, GA 30322, USA; spyu@emory.edu

The Special Issue “Glioblastoma: What Do We Know?”, recently published in *Cells*, focuses on recent progress in both preclinical and clinical research on glioblastoma multiforme (GBM). GBM is the most aggressive and deadly primary brain tumor in human adults, characterized by rapid growth, invasive infiltration into surrounding tissues, and often resistance to conventional cancer therapies. Significant effort from collaborations of around 40 research groups in different countries contributed to six excellent papers in this Special Issue. These papers reported exciting discoveries, informative clinical observations, and innovative experimental therapies against GBM in animal models and human cases.

The review article “Metabolic Reprogramming in Glioblastoma Multiforme: A Review of Pathways and Therapeutic Targets”, authored by Ballen et al. at the Johns Hopkins University School of Medicine, summarizes the immunotherapeutic strategy of metabolic reprogramming of GBM. As widely noted, metabolic reprogramming enables cancer cells to adapt their metabolism to support rapid growth in an oxygen- and nutrient-deficient environment [1]. The metabolic changes include complex and coordinated alterations in cellular and signaling pathways. Addressing the complexity and adaptability of GBM metabolism, this review provides a deeper understanding of metabolic reprogramming and offers predictions for the development of target-specific, more effective therapeutic interventions against GBMs.

In immune responses, T and NK cells have been identified as key players in anti-tumor defense [2]. Inhibitory receptors are critical for regulating the function of immune cells, including T and NK cells. The overexpression of inhibitory receptors on the surfaces of these cells reduces their anti-tumor activities. In the paper “Reduced T and NK Cell Activity in Glioblastoma Patients Correlates with TIM-3 and BAT3 Dysregulation”, Ahmady et al. of Australian and Chinese groups analyzed 11 commonly studied checkpoint and inhibitory receptors. They identified that the expression of HAVCR2 (TIM3) and ENTPD1 (CD39) in the GBM brain was significantly enhanced compared to the expression in the normal brain and lower grade glioma. The cellular surface TIM-3, but not ENTPD1, was elevated on activated CD4+ and CD8+ T cells, as well as on NK cells from GBM patients, compared to T and NK cells from healthy donors. On the other hand, the BAT3 expression significantly decreased in those cells. These pro-inhibitory changes were correlated with low levels of the activation marker CD69 and the pro-inflammatory cytokine IFN in T and NK cells of GBM patients. The authors proposed that the suppression of CD4+, CD8+ T, and NK cells was mediated partly by a dysregulation of TIM-3 and BAT3 expression, which was associated with downstream immunoregulatory and altered functions.

Virotherapy has been a promising approach for oncological diseases, including aggressive brain tumors such as GBM [3]. The work by Ageenko et al. from Russia was reported in the paper “Efficacy of Oncolytic Virus VV-GMCSF-Lact Against Immunocompetent Glioma”. The investigation explored the anti-GBM activity of the recombinant vaccinia virus VV-GMCSF-Lact, focusing on in vivo studies of intravenous and intratumoral viral delivery in a rat model of orthotopically transplanted C6 glioma. Interestingly, only with intratumoral administration of the virus led to a significant decrease in tumor cell proliferation. The anti-tumor toxic effect of VV-GMCSF-Lact was also showed in a mouse GL261 glioma model. These results help to determine the therapeutic viral doses and identify an optimal administration route.

In a clinical report of “CD99 Expression and Prognostic Impact in Glioblastoma: A Single-Center Cohort Study”, Rocca et al. from a collaboration of several Italian groups evaluated the expression and prognostic impact of CD99, which is a membrane glycoprotein involved in cellular migration and invasion [4]. In a cohort of GBM patients who received surgery, radiotherapy, and temozolomide, a retrospective analysis indicated that CD99 expression was not associated with overall survival, regardless of the assessment method applied. This study suggests that CD99 expression levels in GBM cells do not significantly affect survival. Further basic and clinical research is warranted to confirm the interesting observation made with a relatively small sample size of 46 patients.

The paper “Upregulation of the Renin–Angiotensin System Is Associated with Patient Survival and the Tumour Microenvironment in Glioblastoma” is published by Lozinski et al. of several Australian groups, which provides new information on the relationship between the renin-angiotensin system (RAS) in GBM and patients’ survival. The RAS is a key regulator of cardiovascular homeostasis [5]. Recent research has suggested that RAS activation and inhibition are associated with various forms of cancer. RAS blockage may confer a protective effect in some cancers, while the process may also have negative or even adverse effects. It has been assumed that differences in receptors and effectors may account for these findings. In this Special Issue paper, the authors examined the RAS genes in 12 patient-derived glioblastoma cell lines that received chemoradiation. The data revealed that high AGTR1 expression was independently associated with worse progression-free survival (PFS). The combined expression of RAS receptors ATP6AP2, AGTR1, and AGTR2 was positively linked with gene pathways in hypoxia, microvasculature, stem cell plasticity, and the molecular characterization of glioblastoma subtypes. Interestingly, ATP6AP2 and AGTR1 were upregulated after chemoradiotherapy and correlated with increased HIF1A expression. Results from this clinical study are valuable for a better understanding of how specific RAS genes may correlate with the tumor microenvironment and GBM survival outcomes.

Another exciting report is from Jiang et al. at Emory University in the USA. In the paper entitled “Reprogramming Glioblastoma Cells into Non-Cancerous Neuronal Cells as a Novel Anti-Cancer Strategy”, the authors provide in vitro and in vivo data of a novel gene therapy of direct reprogramming of GBM cells, which directly converts proliferating GBM cells to postmitotic neuronal cells, coined induced neurons (iNeurons) [6]. The unique feature of this novel approach is that the induced neuronal conversion bypasses the stem cell stage. This direct conversion was achieved through viral delivery of NeuroD1 (ND1), a master transcriptional factor of neurogenesis [7]. In cultured human GBM cell lines of temozolomide (TMZ)-sensitive and TMZ-resistant cells, ND1-expressing cells displayed neuronal markers MAP2, TUJ1, and NeuN. A novel discovery is that ND1-induced transdifferentiation was sensitive to Wnt signaling regulation and was markedly augmented under hypoxic conditions. Converted cells showed reduced proliferation and diminished migration, with marked increases in cell death. Strikingly, converted cells expressed the anti-tumor gene p53, which is normally detected only in non-cancer cells. The anti-tumor action of the gene therapy was validated in an orthotopic GBM mouse model. Thus, direct reprogramming and direct conversion can overcome drug resistance, converting heterogeneous malignant GBM cells into normal neuron-like cells.

Collectively, the Special Issue provides a rich source of the most recent progress in the investigation of the underlying mechanisms, therapeutic strategies, and clinical significance of GBM research. Further collaborative efforts and innovative approaches should be encouraged for the development of effective anti-GBM therapies.
